# Multimodal DeepSurv model with SHAP-based interpretation for predicting local failure in patients with brain metastases undergoing hypofractionated stereotactic radiotherapy

**DOI:** 10.3389/fnins.2026.1908548

**Published:** 2026-08-12

**Authors:** Long Jin, Qifan Zhao, Ying Xiao, Yuan Hu, Jie Wu, Gaofei Zhang

**Affiliations:** 1 Department of Radiation Oncology, Shaanxi Provincial People's Hospital, Xi'an, China; 2 School of Computing and Data Science, The University of Hong Kong, Hong Kong, China

**Keywords:** brain metastases, explainable AI, MRI, multimodal, radiomics

## Abstract

**Objective:**

Local Progression-Free Survival (LPFS) is an important clinical endpoint following hypofractionated stereotactic radiotherapy (HSRT) for brain metastases (BMs). However, accurate prediction of local failure risk remains challenging because conventional clinical factors do not fully capture tumor heterogeneity. Therefore, we aimed to develop and validate an explainable radiomics-based deep learning survival model to predict LPFS after HSRT and identify patients at elevated risk of local failure.

**Methods:**

This retrospective study included 100 patients with BMs treated with HSRT between 2019 and 2023 as training dataset, while utilized medical images and clinical features from 40 patients from publicly available dataset PROTEAS project. Radiomic features were extracted from the contrast-enhancing BM (T1-weighted contrast-enhanced MRI) and edema (T2-FLAIR MRI). A baseline Cox proportional hazards model incorporating clinical variables was established for comparison. Three DeepSurv models were developed using different combinations of clinical, BM-derived, and edema-derived radiomic features. Model performance was evaluated using the concordance index (C-index). Kaplan–Meier analysis was performed for risk stratification, and Shapley Additive Explanations (SHAP) were used to identify the most influential prognostic features.

**Results:**

The best predictive performance was achieved by the DeepSurv model integrating clinical, BM-derived, and edema-derived radiomic features, outperforming the clinical-only Cox model (C-index: 0.61 vs. 0.72). The model effectively stratified patients into distinct prognostic groups with significantly different local failure-free survival outcomes (*P* = 0.002). SHAP analysis identified edema Gray Level Co-occurrence Matrix (GLCM) Cluster Prominence as the most important predictor of local failure, followed by edema Large Area High Gray Level Emphasis (LAHGLE), tumor GLCM Cluster Prominence, and tumor LAHGLE. Patients with high edema GLCM Cluster Prominence exhibited significantly worse local failure-free survival than those with low values (*P* = 0.0155).

**Conclusion:**

A combination of clinical, tumor-derived, and edema-derived radiomic features predicted Local Progression-Free Survival more accurately than clinical variables alone. Peritumoral edema features contributed more strongly to local failure prediction than tumor features, highlighting the prognostic importance of the tumor microenvironment. Patients received targeted therapy with low-risk radiomic profiles benefit from HSRT for local control.

## 1 Introduction

It is estimated that 10%–40% of patients with solid tumors will develop BMs during the course of their disease, with an incidence that may be more than ten times higher than that of primary malignant brain tumors (Lamba et al., 2021). With continuous advances in imaging technologies and prolonged survival of patients with malignant tumors, the incidence of BMs has increased compared with previous years. However, due to incomplete registration records of secondary malignancies, accurate epidemiological data on BMs are lacking both domestically and internationally. A previous study (Vogelbaum et al., 2022) reported that the annual incidence of BMs in the United States ranges from 70,000 to 400,000 cases. Lung cancer, breast cancer, melanoma, renal cell carcinoma, and colorectal cancer are the major primary malignancies responsible for BMs. Evidence suggests that the incidence of BMs may continue to rise.

Patients with BMs generally have a poor prognosis, with a median overall survival ranging from several weeks to a few months in untreated patients. Radiotherapy is an effective treatment for BMs, capable of controlling tumor growth, alleviating symptoms, and prolonging survival. The three major radiotherapy approaches for BMs include whole-brain radiotherapy (WBRT), single-fraction stereotactic radiosurgery (SRS), and hypofractionated stereotactic radiotherapy (HSRT). WBRT was historically the primary treatment for patients with multiple BMs. However, due to the adverse effects associated with WBRT, such as fatigue and cognitive decline, treatment strategies have gradually shifted toward HSRT and SRS (Vogelbaum et al., 2022; Garsa et al., 2021). Among these approaches, HSRT is more consistent with the classical radiobiological “4R” principles (repair, reassortment, repopulation, and reoxygenation). HSRT can deliver a higher cumulative dose to larger target volumes while minimizing the risk of toxicity, although the optimal dose and fractionation scheme have not yet been fully established. HSRT is commonly applied to BMs with diameters >2–3 cm and/or lesions located in eloquent regions such as the brainstem, optic nerve, optic chiasm, and internal capsule. The most commonly used fractionation regimen is 52–52.5 Gy delivered in 13–15 fractions. In cases involving both large and small volume lesions, treatment using a single isocenter with the dose prescribed according to the higher fractionation schedule may also be considered (Garsa et al., 2021; Pikis et al., 2025; Myrehaug et al., 2022; Fatima et al., 2019).

Local control (LC) assessment of BMs is an important clinical endpoint. Patients with stable disease after treatment are classified as achieving LC, whereas those with disease progression are categorized as having local failure (LF) (Lin et al., 2015). Early identification of LF following radiotherapy is critical for timely modification of treatment strategies, ensuring that patients receive the most effective therapy and maximizing their chances of favorable outcomes. Currently, several clinical prognostic indicators, including Karnofsky Performance Status (KPS), patient age, tumor volume, presence of extracranial metastases, and the number of BMs, have been used to construct prognostic indices for predicting patient survival. However, due to the substantial heterogeneity in treatment outcomes observed in clinical practice, no precise predictive model for radiotherapy response in BMs has yet been established.

Driven by advances in artificial intelligence (AI), radiomics has demonstrated considerable potential in improving cancer diagnosis and prognosis, either independently or in combination with other clinical information. These radiomic features can serve as valuable inputs for developing deep learning models aimed at predicting treatment response or local control in patients with BMs. Given the large amount of MRI data routinely acquired as part of standard clinical care for patients with BMs, radiomics offers a promising, efficient, and noninvasive approach for characterizing metastatic brain tumors and predicting treatment outcomes. This study aimed to develop a pre-therapeutic radiomics-based deep learning survival model to predict Local Progression-Free Survival (LPFS) following HSRT in patients with BMs. The model was further evaluated for risk stratification and clinical utility. In addition, Shapley value-based explainable AI was applied to identify globally important features and derive interpretable recommendations for selecting BMs patients may benefit most from HSRT.

## 2 Methods

### 2.1 Eligibility criteria and patient information

This retrospective training cohort study collected imaging data and relevant clinical information from 100 patients clinically diagnosed with BMs at Shaanxi Provincial People's Hospital between June 2019 and June 2023. The study was approved by the institutional ethics committee (Approval No. 2023K-S004).The external validation cohort comprised 40 patients from the publicly available PROTEAS project (Flouri et al., 2025), a longitudinal dataset of patients with brain metastases. The dataset includes pretreatment MRI imaging studies acquired from 40 patients with manually segmented brain metastases. Manual segmentations of the enhancing tumor and peritumoral edema were provided for radiomics feature extraction and external validation.

The inclusion criteria were as follows: (1) age >18 years; (2) extracranial solid malignancy confirmed by pathology; (3) BMs identified on contrast-enhanced brain MRI; (4) no prior surgical resection or radiotherapy for BMs, while systemic therapy was permitted; (5) stable and controllable extracranial disease without radiological progression within the previous 3 months; and (6) an expected survival time of at least 3 months. The exclusion criteria included: (1) previous cranial radiotherapy; (2) leptomeningeal metastasis or concurrent leptomeningeal dissemination; (3) primary tumors of small-cell lung cancer, lymphoma, or multiple myeloma; (4) severe concomitant systemic diseases; (5) inability to undergo contrast-enhanced brain MRI examination; and (6) incomplete follow-up data. The training and validation datasets were also randomly divided in a 7:3 ratio. All study procedures were conducted in accordance with the principles of the Declaration of Helsinki. All procedures complied with applicable regulatory requirements and institutional guidelines. Prior to review, all patient medical records were anonymized to remove identifiable information.

For patients receiving HSRT, treatment plans were developed based on contrast-enhanced MRI images. Patients were immobilized in the supine position using a thermoplastic mask. Contrast-enhanced brain MRI images were fused with planning CT images for target delineation. The gross tumor volume (GTV) was defined as the radiologically visible brain metastasis, excluding the surrounding edema. A 3-mm isotropic margin was added to the GTV to generate the planning target volume (PTV). Treatment plans were generated using the Monaco or Pinnacle treatment planning systems. The dose distribution was optimized such that the 95%–105% isodose lines covered the PTV. HSRT was delivered using either intensity-modulated radiotherapy (IMRT) or volumetric modulated arc therapy (VMAT). The prescribed dose was 52 Gy in 13 fractions (4.0 Gy per fraction) or 52.5 Gy in 15 fractions (3.5 Gy per fraction). Treatment was delivered using a Varian 21EX or Elekta Infinity linear accelerator with 6-MV X-rays. During radiotherapy, dehydration therapy was administered as clinically indicated to control cerebral edema. Patients with a history of seizures received prophylactic antiepileptic medication.

### 2.2 Imaging data and ROI segmentation

MRI examinations were performed using a 3.0-T scanner equipped with a 32-channel phased-array head coil. The imaging protocol included axial T1-weighted imaging (T1WI), axial T2-weighted imaging (T2WI), fluid-attenuated inversion recovery (FLAIR), sagittal T2WI, and contrast-enhanced T1-weighted imaging in the axial, sagittal, and coronal planes. The scanning parameters were as follows: T1WI and contrast-enhanced T1WI, repetition time (TR) = 200 ms and echo time (TE) = 12 ms; T2WI, TR = 3,900 ms, and TE = 120 ms; FLAIR, TR = 7,600 ms and TE = 170 ms. All sequences were acquired with a slice thickness of 3 mm, an interslice gap of 1 mm, and a field of view (FOV) of 256 mm × 256 mm. MRI images of all patients were exported from the Picture Archiving and Communication System (PACS) workstation in Digital Imaging and Communications in Medicine (DICOM) format. Radiomic features were extracted from the contrast-enhancing BM (T1-CE MRI sequence) and the surrounding edema (T2-FLAIR sequence)

The BM and peritumoral edema regions were manually segmented as separate regions of interest (ROIs) on MRI by radiologist L.J., with 10 years of experience.

### 2.3 Image preprocessing and feature extraction

Radiomic features were extracted using PyRadiomics according to the Image Biomarker Standardization Initiative (IBSI) recommendations. Prior to feature extraction, all images and corresponding segmentation masks were resampled to an isotropic voxel spacing of 1 × 1 × 1 mm^3^, and gray-level intensities were discretized using a fixed bin width of 5. A total of 79 handcrafted radiomic features were extracted independently from both the tumor and peritumoral edema regions, including shape, first-order intensity, and texture features (GLCM, GLRLM, GLSZM, GLDM, and NGTDM) computed from the original images. Rather than performing outcome-driven feature selection, the complete predefined radiomic feature set was used as input to the DeepSurv model to preserve complementary morphological and textural information from both regions.

### 2.4 Explainable radiomics machine learning model design

To predict LPFS, a baseline prognostic model was first established using existing clinical prognostic indices, including The Karnofsky Performance Status (KPS) score and Targeted Therapy. The performance of these conventional clinical models served as a reference for evaluating the additional prognostic value of radiomic features.

Subsequently, three deep learning-based survival models were developed using different combinations of radiomic and clinical features. The first model incorporated radiomic features extracted from the brain metastasis (BM) region together with clinical characteristics. The second model utilized radiomic features derived from the peritumoral edema region combined with clinical characteristics. The third model integrated radiomic features from both the BM and edema regions along with clinical variables. Each neural network was trained to learn the relationship between the input features and LPFS outcomes and to generate an individualized prognostic risk score.

The proposed DeepSurv model was trained using the Adam optimizer to update the network parameters over multiple epochs. Hyperparameter optimization was performed using random search. The search space included the dropout rate (0.2–0.6), number of hidden layers (1–7), and the number of neurons in each hidden layer (5–90). The optimal hyperparameter configuration consisted of five hidden layers with 52, 69, 53, 29, and 20 neurons, respectively, a dropout rate of 0.587, and a learning rate of 0.00801. The hidden layers employed the rectified linear unit (ReLU) activation function to introduce nonlinearity, enabling the network to learn complex relationships between multimodal input features and patient survival risk.

To further assess risk stratification capability, patients in the training cohort were categorized into low- and high-risk groups according to the 50th percentile values of the predicted continuous risk scores. The same thresholds derived from the training cohort were subsequently applied to the independent test cohort to assign patients to the corresponding risk groups. Kaplan–Meier survival analysis was then performed to compare LPFS among the stratified groups. The model demonstrating the best prognostic performance was selected for further evaluation and interpretation.

**Figure 1:**
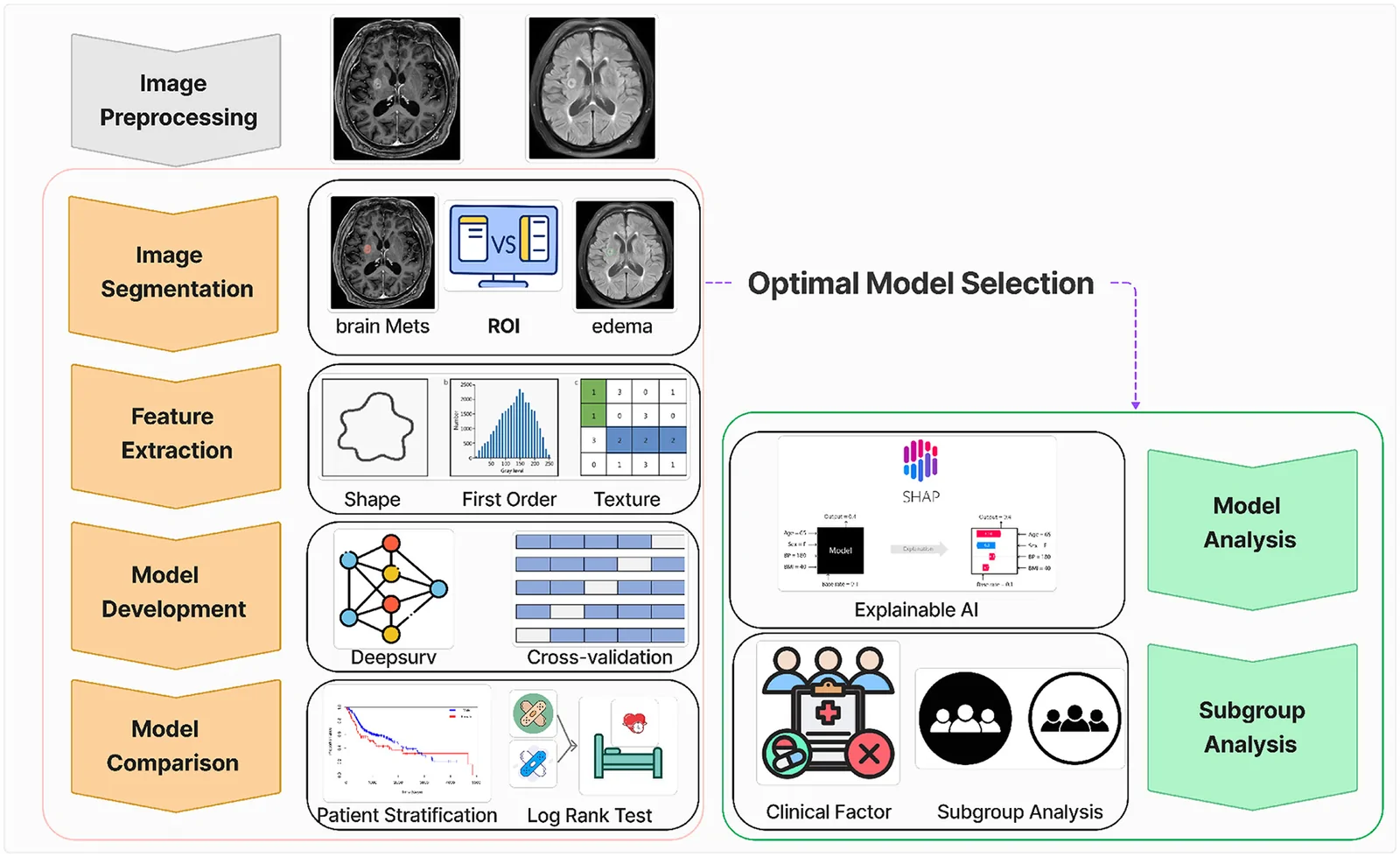
Diagram of the training and analyzing procedure.

To improve model interpretability, Shapley Additive Explanations (SHAP) were employed to quantify the contribution of individual features to the model predictions. Global feature importance was assessed by calculating the mean absolute SHAP value of each feature across the study cohort, and features were ranked according to their relative importance. SHAP summary plots were generated to visualize the influence of the most informative features on the predicted risk of local failure. The overall study workflow is illustrated in Figure 1.

### 2.5 Statistical analysis and software

Statistical analyses were conducted using Python 3.8 (Python Software Foundation, Wilmington, DE, USA). Deep learning-based survival models were developed and trained using PyTorch. Survival analyses, including Kaplan–Meier estimation, log-rank testing, and concordance index (C-index) calculation, were performed using the Lifelines package.

Model discrimination was assessed using the C-index, which measures the agreement between predicted risk scores and observed survival outcomes. To evaluate the prognostic utility of the proposed models, Kaplan–Meier survival curves were generated for the stratified risk groups and compared using the log-rank test. All statistical tests were two-sided, and a *p*-value less than 0.05 was considered statistically significant.

## 3 Results

### 3.1 Patient baseline characteristics

The study comprised 100 patients with complete clinical and radiomic data. The baseline characteristics of the cohort are summarized in Table 1. The median follow-up duration is 11 month. Regarding lesion distribution, the parietal lobe was the most common lesion location, accounting for 32.0% of patients, followed by the cerebellum (21.0%) and occipital lobe (18.0%). In terms of the primary tumor origin, lung cancer represented the predominant source of BMs, comprising 62.0% of cases, whereas breast cancer and other malignancies accounted for 20.0% and 18.0%, respectively. With respect to functional status, a Karnofsky Performance Status (KPS) score of 80 was the most frequently observed category (31.0%), followed by scores of 70 (26.0%), 60 (21.0%), and 90 (21.0%). Furthermore, targeted therapy was administered to 21.0% of patients, while 79.0% did not receive targeted treatment. Regarding the local failure endpoint, the time-to-event intervals exhibited considerable variability across patients, ranging from 3 to 48 intervals. The most frequently observed interval was 6 (17.0%), followed by 8 (10.0%), indicating substantial heterogeneity in the timing of local failure events within the study population.

**Table 1:** Main baseline clinical characteristics of patients.

Characteristic	Dataset, no. (%)	
	Training (*n* = 100)	Testing (*n* = 43)
Lesion location		
Frontal lobe	14 (14.0)	10 (25.0)
Temporal lobe	9 (9.0)	5 (12.5)
Parietal lobe	32 (32.0)	11 (27.5)
Occipital lobe	18 (18.0)	4 (10.0)
Cerebellum	21 (21.0)	9 (22.5)
Brainstem	5 (5.0)	0 (0.0)
Other	1 (1.0)	1 (2.5)
Primary tumor site		
Lung	62 (62.0)	26 (65.0)
Breast	20 (20.0)	14 (35.0)
Other	18 (18.0)	0 (0.0)
KPS score		
50	1 (1.0)	2 (5.0)
60	21 (21.0)	0 (0.0)
70	26 (26.0)	8 (20.0)
80	31 (31.0)	18 (45.0)
90	21 (21.0)	11 (27.5)
100	0 (0.0)	1 (2.5)
Targeted therapy		
No	79 (79.0)	35 (87.5)
Yes	21 (21.0)	5 (12.5)

### 3.2 Survival feature importance

**Figure 2:**
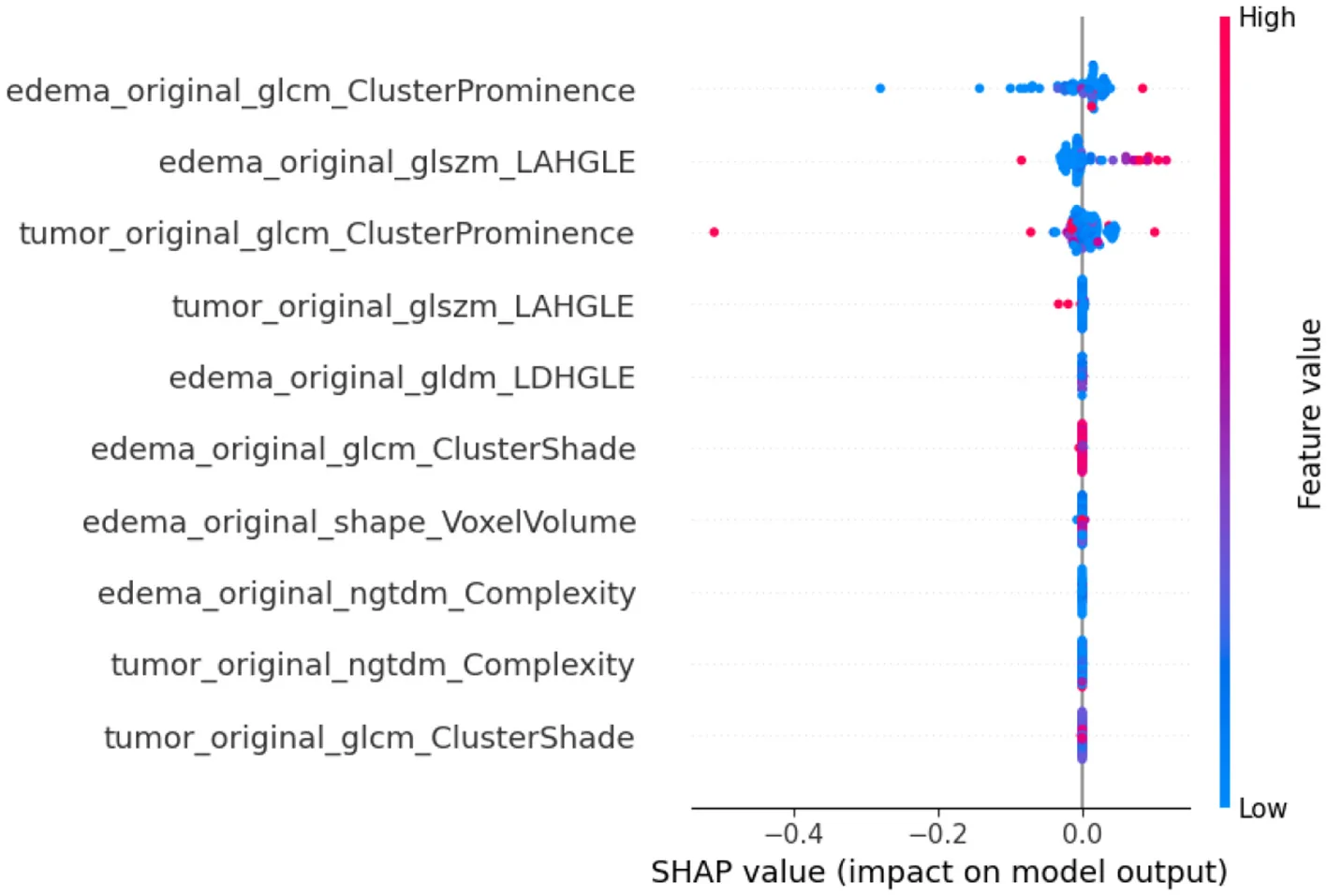
SHAP summary plot of the DeepSurv model for local failure prediction. The features are ranked according to their mean absolute SHAP values, representing their overall contribution to the model output. The *x*-axis shows the SHAP value, indicating the impact of each feature on the predicted hazard of local failure, while the *y*-axis lists the top 10 radiomic features. Each point represents an individual patient, and the color denotes the feature value, ranging from low (blue) to high (red).

The model and patient radiomics data were used to calculate global feature importance using SHAP values (Figure 2). Among the top four predictors of local failure, two features were derived from both peritumoral edema and BM region, Gray Level Co-occurrence Matrix (GLCM) Cluster Prominence, Large Area High Gray Level Emphasis (LAHGLE). Overall, edema-derived radiomic features demonstrated greater importance than tumor-derived features, suggesting that the peritumoral region contributed substantially to local failure risk stratification.

Edema GLCM Cluster Prominence was identified as the most important feature, followed by edema LAHGLE, tumor GLCM Cluster Prominence, and tumor LAHGLE. SHAP analysis showed that higher values of edema GLCM Cluster Prominence were generally associated with positive SHAP values, indicating a greater contribution to the predicted hazard of local failure. Similarly, higher values of edema LAHGLE tended to increase the hazard of local failure and were associated with elevated risk scores. Tumor GLCM Cluster Prominence demonstrated a comparable trend, with larger feature values contributing positively to the predicted hazard. A similar, although less pronounced, pattern was observed for tumor LAHGLE. Collectively, these results suggest that increased texture heterogeneity and larger high-intensity homogeneous regions in both the edema and tumor regions were associated with higher predicted local failure risk according to the optimal model.

### 3.3 Survival analysis for subgroups

To investigate whether the most important radiomic feature identified by the optimal model could further stratify patient outcomes, subgroup analysis was performed using edema GLCM Cluster Prominence. Based on the optimal cutoff value of 461,148,989.399045, patients who received HSRT and targeted therapy were divided into high- and low-value subgroups.

**Figure 3:**
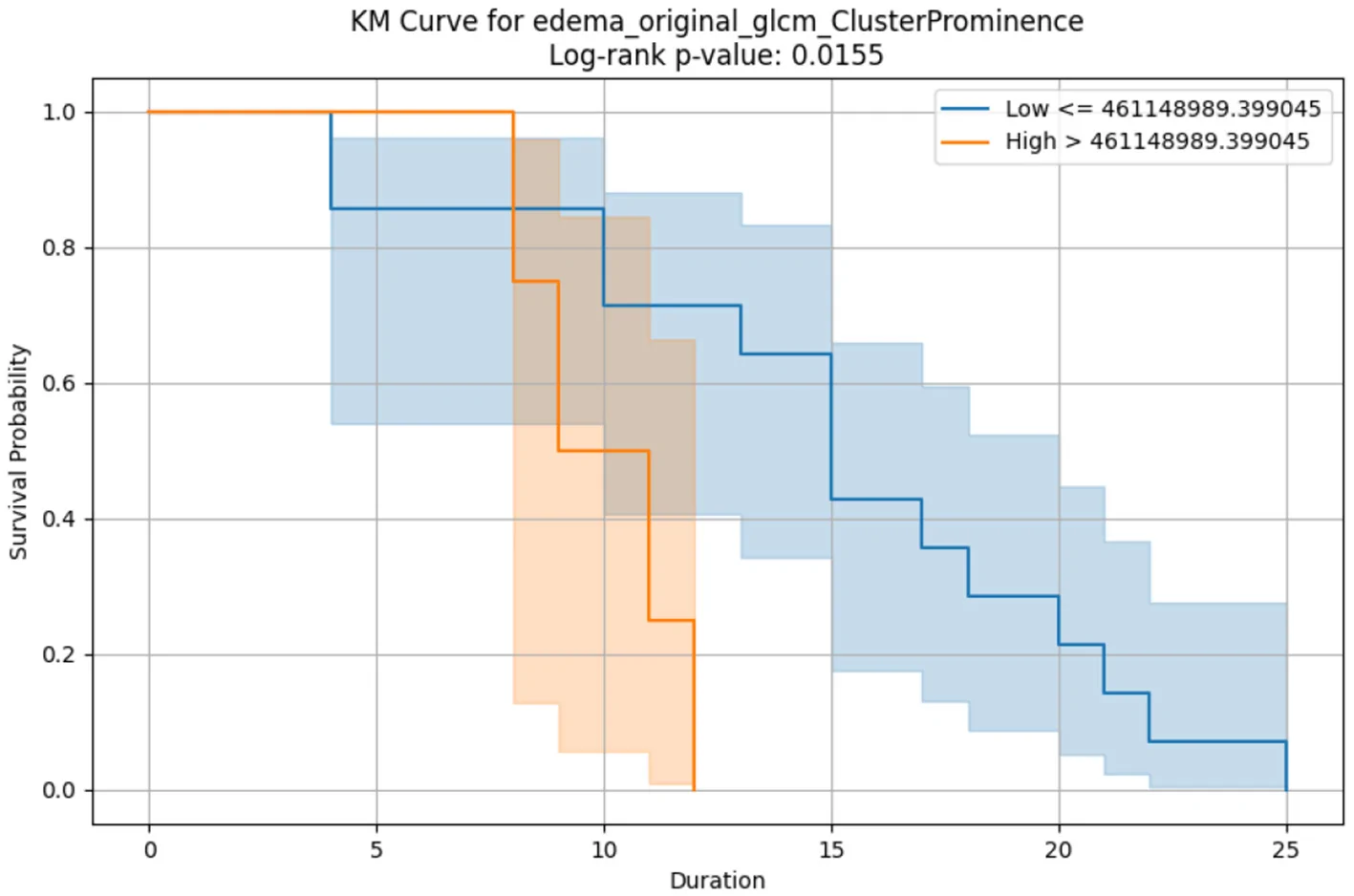
Kaplan–Meier survival curves stratified by edema Gray Level Co-occurrence Matrix (GLCM) Cluster Prominence. The high-value group exhibited significantly poorer local failure-free survival compared with the low-value group (log-rank *p* = 0.0155). Shaded regions represent the 95% confidence intervals of the Kaplan–Meier estimates.

Kaplan–Meier analysis demonstrated a significant separation between the two subgroups (log-rank *p* = 0.0155; Figure 3). Patients with edema GLCM Cluster Prominence values above the cutoff exhibited significantly worse local failure-free survival compared with those in the low-value subgroup. Consistent with the global SHAP analysis, higher values of edema GLCM Cluster Prominence were associated with increased predicted hazard, indicating a greater risk of local failure despite receiving the same treatment regimen. Notably, all patients included in this subgroup analysis underwent HSRT in combination with targeted therapy. Therefore, the observed survival difference suggests that edema GLCM Cluster Prominence can further stratify prognosis within an otherwise clinically homogeneous treatment population. Patients in the low-value subgroup achieved more favorable local control outcomes, whereas patients in the high-value subgroup remained at elevated risk of local failure.

### 3.4 Patient stratification

**Figure 4:**
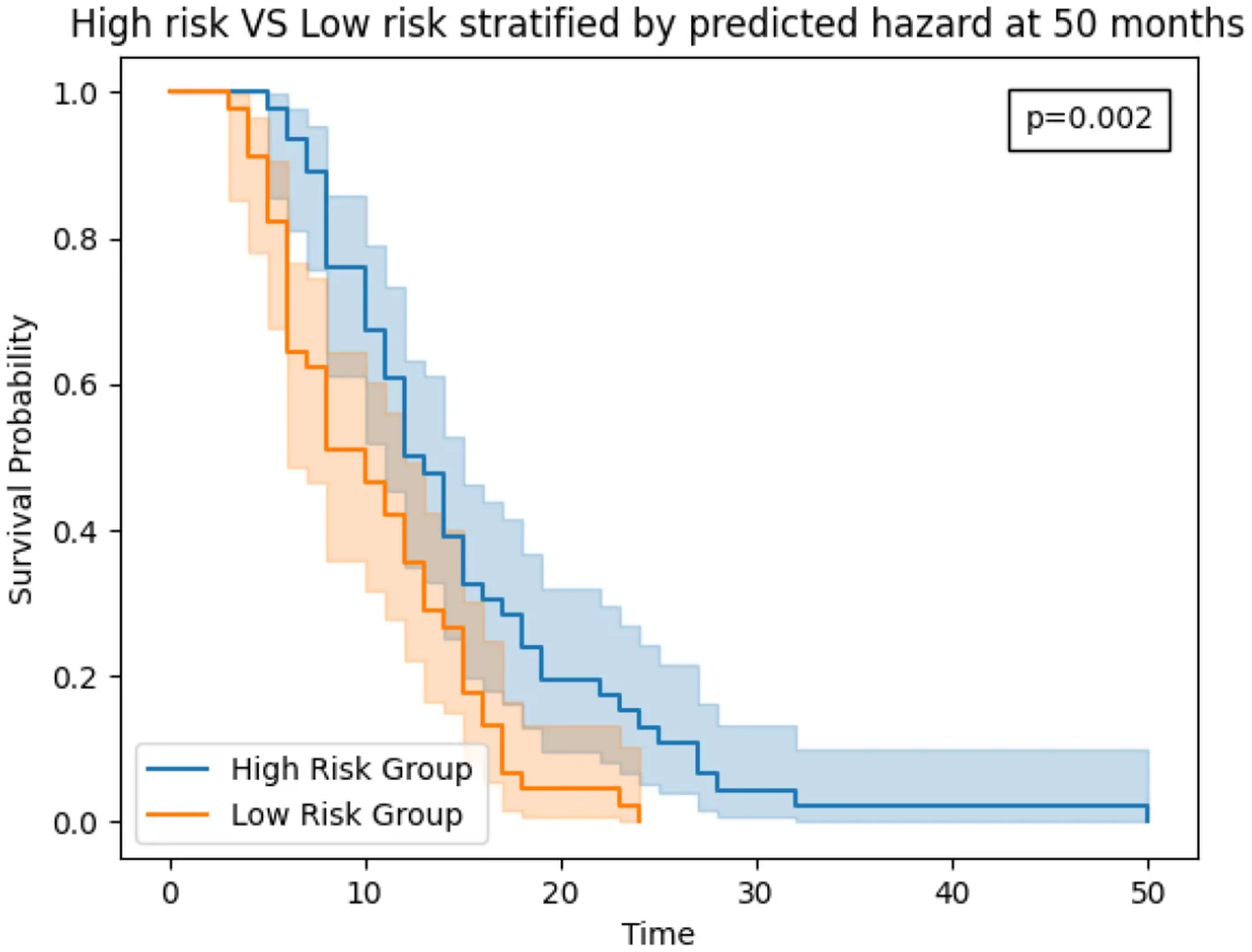
Kaplan–Meier analysis of local failure-free survival for patients stratified by the DeepSurv-predicted risk score. Patients were categorized into high- and low-risk groups using the median predicted hazard as the cutoff. A significant difference in local failure-free survival was observed between the two groups (log-rank *p* = 0.002).

Patients were stratified into high- and low-risk groups according to the predicted hazard score generated by the DeepSurv model, using the median value (50th percentile) as the cutoff. Kaplan–Meier analysis demonstrated a significant difference in local failure-free survival between the two groups (log-rank *p* = 0.002; Figure 4). Patients in the high-risk group exhibited poorer local failure-free survival than those in the low-risk group. The separation of the survival curves indicates that the DeepSurv-derived risk score effectively stratified patients into distinct prognostic groups with different local failure risks.

### 3.5 Model comparison and performance

To establish a clinical baseline for comparison, we first evaluated a Cox proportional hazards model incorporating Lesion Location, KPS Score, Targeted Therapy, and 3D diameter of BM. The predictive performances of the baseline Cox model and the DeepSurv models are summarized in Table 2.

**Table 2:** Performance of the survival models to predict hazard rate.

Model	Cross validation	External validation
	Concordance index mean	Concordance index
Clinical feature	0.63 (0.61–0. 66)	0.59 (0.56–0. 61)
Clinical, BM-based radiomic feature	0.69 (0.64–0. 71)	0.64 (0.62–0. 67)
Clinical, edema-based radiomic feature	0.67 (0.63–0. 69)	0.64 (0.61–0. 66)
Clinical, BM and edema-based radiomic feature	0.73 (0.69–0. 76)	0.72 (0.69–0. 74)
ResNet-50, EfficientNet-B0, and MLP	0.69 (0.66–0. 71)	0.68 (0.66–0. 72)

The baseline Cox model achieved a C-index of 0.61 and 0.63 in the training and validation cohorts, respectively. To investigate the added prognostic value of radiomics, three DeepSurv models were subsequently developed by integrating clinical features with different radiomic feature sets. The DeepSurv model combining clinical and BM-based radiomic features achieved C-indices of 0.69 and 0.70 in the training and validation cohorts, respectively. Similarly, the DeepSurv model incorporating clinical and edema-based radiomic features yielded C-indices of 0.67 and 0.71. Among all evaluated models, the DeepSurv model integrating clinical, BM-based, and edema-based radiomic features demonstrated the best predictive performance, achieving a C-index of 0.73 in the training cohort and 0.72 in the validation cohort. Compared with the baseline Cox model, the combined DeepSurv model improved the C-index by 0.12 in the training cohort and 0.11 in the validation cohort. The reproduced ResNet-based multimodal (Volovăṭ et al., 2025) model achieved a C-index of 0.69.

## 4 Discussion

This study developed a radiomics-based DeepSurv model to predict LPFS after HSRT in patients with BMs. The combined model integrating clinical, brain metastasis, and edema radiomic features achieved the best prognostic performance and outperformed the baseline clinical Cox model. SHAP analysis further demonstrated that edema-derived features contributed more strongly to local failure prediction than tumor-derived features. Furthermore, the model output successfully stratified patients into high-risk and low-risk groups with significantly different local failure-free survival outcomes. In addition, edema Gray Level Co-occurrence Matrix (GLCM) Cluster Prominence, the most important radiomic feature identified by SHAP analysis, effectively distinguished patient subgroups with different prognoses.

Our finding indicates integrating radiomic features with clinical variables substantially improved prognostic performance—achieving a C-index of 0.72 vs. 0.59 for clinical factors alone—demonstrates that radiomics adds meaningful value in predicting the hazard of local failure following HSRT. This aligns closely with Buchner et al. (2024), who reported that a combined radiomic-clinical model (C-index 0.77) outperformed clinical-only models (C-index 0.70) for predicting local failure risk, with high-risk patients showing 74% failure at 24 months compared with only 9% in low-risk patients. Similarly, Mouraviev et al. (2020) found that adding radiomic features to clinical variables increased the AUC from 0.669 to 0.793—a 19% improvement—indicating substantially better discrimination of patients at elevated hazard. Together, these three studies provide consistent evidence that radiomic features, particularly those capturing tumor and peritumoral heterogeneity, significantly enhance the prediction of local failure hazard beyond what clinical variables alone can achieve, supporting their integration into risk stratification models for BMs patients undergoing stereotactic radiotherapy. Beyond the observed improvement in predictive performance, the proposed DeepSurv-mutlimodal framework offers several methodological advantages over conventional survival models and recent radiomics-based machine learning approaches. First, unlike traditional Cox proportional hazards models, DeepSurv is capable of modeling complex nonlinear interactions between clinical variables and radiomic features, which are likely to better reflect the biological heterogeneity of brain metastases. Second, our framework integrates radiomic information from both the enhancing tumor and the surrounding peritumoral edema. While most previous studies have focused primarily on the tumor itself, our results demonstrate that edema-derived features contributed more strongly to local failure prediction, highlighting the importance of the tumor microenvironment.

SHAP analysis revealed that edema-derived radiomic features contributed more strongly to local failure prediction than tumor-derived features, suggesting that the peritumoral edema region contains clinically relevant prognostic information beyond the enhancing tumor alone. This observation is consistent with previous studies indicating that the peritumoral region may contain imaging characteristics associated with biological processes related to radioresistance, including hypoxia, angiogenesis, and tumor-microenvironment interactions (Youssef et al., 2024). Furthermore, Berghoff et al. (2013) reported that the extent of peritumoral edema was independently associated with prognosis and tumor invasiveness in patients with brain metastases. In our study, higher edema GLCM Cluster Prominence was associated with an increased predicted risk of local failure, whereas lower values were associated with a reduced risk. GLCM Cluster Prominence quantifies texture asymmetry, and higher values indicate greater structural heterogeneity within the peritumoral edema region. A recent systematic review further identified peritumoral edema texture features, including heterogeneity-related metrics, as important imaging biomarkers for predicting treatment outcomes in patients with brain metastases undergoing stereotactic radiosurgery (Desai et al., 2026). Collectively, these findings suggest that increased heterogeneity within the edema region may provide imaging biomarkers associated with an unfavorable tumor phenotype. However, it should be emphasized that the SHAP analysis identifies the contribution of individual features to model predictions and does not establish biological causality. Therefore, the proposed biological interpretation linking edema-derived radiomic features to hypoxia, angiogenesis, and the tumor microenvironment should be regarded as a hypothesis supported by previous literature rather than direct evidence of the underlying mechanisms. Further studies integrating radiomic, histopathological, and molecular data are warranted to validate these potential biological associations.

Our DeepSurv model integrating clinical, BM-based, and edema-based radiomic features achieved a C-index of 0.72 in the validation cohort, outperforming the clinical-only model (C-index 0.63) and demonstrating competitive performance relative to published reports. Compared with traditional machine learning approaches such as the elastic net model by Buchner et al. (2024) (C-index 0.77) and the random forest classifier by Mouraviev et al. (2020) (AUC improvement from 0.669 to 0.793 after adding radiomics), our deep learning-based DeepSurv model offers the advantage of capturing complex non-linear relationships between radiomic features and survival outcomes without requiring manual feature selection. Furthermore, unlike most prior studies that focused on single-fraction SRS or postoperative cavities, our model is specifically tailored to HSRT for intact BMs, addressing an understudied clinical scenario. The superior contribution of edema-derived features in our SHAP analysis, which aligns with biological evidence linking peritumoral heterogeneity to hypoxia-mediated radioresistance (Yavorska et al., 2024; Akhavan-Sigari et al., 2025).

Another important consideration is the clinical heterogeneity of the study population. Our cohort included patients with brain metastases originating from different primary tumor types, predominantly lung and breast cancer, as well as patients receiving different systemic treatment strategies, including targeted therapy. These factors are well-established determinants of tumor biology, treatment response, and prognosis, and may therefore influence both radiomic characteristics and the predictive performance of survival models. Although primary tumor site and targeted therapy were incorporated as clinical variables in the proposed multimodal framework, other clinically relevant factors, such as molecular alterations and the specific type and timing of systemic therapies, were not consistently available in this retrospective cohort and, therefore, could not be included (Yang et al., 2026a,b,c, 2024). Consequently, the identified radiomic signature should be interpreted as reflecting prognostic imaging patterns within this heterogeneous population rather than as a tumor type-specific biomarker. Nevertheless, the favorable performance observed on the independent external validation cohort suggests that the proposed framework captures imaging characteristics that are reasonably robust across different patient populations. Future multicenter studies with larger cohorts and more detailed molecular and treatment information are warranted to further evaluate the stability and generalizability of the proposed radiomic signature across specific tumor subtypes and therapeutic strategies.

In conclusion, this study demonstrates that integrating clinical variables with tumor- and edema-derived radiomic features within a DeepSurv framework improves the prediction of local failure following hypofractionated stereotactic radiotherapy for brain metastases. The SHAP analysis further provides insight into the relative contribution of individual clinical and radiomic features, enhancing the interpretability of the proposed model. Nevertheless, this study has several limitations, including its retrospective single-center design, limited sample size, and the lack of detailed molecular and treatment-specific information. Although external validation demonstrated encouraging model generalizability, further validation in larger multicenter cohorts with standardized imaging protocols and comprehensive molecular profiling is warranted before clinical implementation.

## Data Availability

The datasets analyzed for this study can be found in the https://github.com/snowflake-Zhao/BrM_HSRT.
